# Human Adenovirus Type 7 Outbreak in Police Training Center, Malaysia, 2011

**DOI:** 10.3201/eid1805.110865

**Published:** 2012-05

**Authors:** Mohd Apandi Yusof, Tengku Rogayah Tengku Abdul Rashid, Ravindran Thayan, Khairul Azuan Othman, Norhasnida Abu Hasan, Norfaezah Adnan, Zainah Saat

**Affiliations:** Institute for Medical Research, Kuala Lumpur, Malaysia

**Keywords:** viruses, human adenovirus type 7, adenovirus, acute respiratory disease, Malaysia, HAdV-7, human adenovirus B, human adenovirus C, polymerase chain reaction, sequencing, hexon gene

## Abstract

In March 2011, an outbreak of acute respiratory disease was reported at the Kuala Lumpur (Malaysia) Police Training Centre. Approximately 100 trainees were hospitalized and 5 were admitted to the intensive care unit. Three of these 5 trainees died. Human adenovirus type 7 was identified as the etiologic agent.

Human adenoviruses (HAdVs) consist of nonenveloped, double-stranded DNA and belong to the family *Adenoviridae*, genus *Mastadenovirus*. The 51 recognized serotypes of human adenoviruses have been placed in 7 human adenovirus species, A–G (*1*). These viruses cause infections ranging from mild syndromes to severe, life-threatening disease.

Depending on the species, these viruses may infect respiratory, conjunctival, gastrointestinal, and genitourinary sites. They have been recognized for decades as the primary causes of acute respiratory disease (ARD), gastrointestinal infection, and fever (*2*). Outbreaks of adenoviruses associated with respiratory disease have been reported worldwide (*3**,**4*) and commonly occur among the military trainees (*5**,**6*). These cases of ARD are most frequently associated with a strain of HAdV-B, HAdV-7 (*7*).

We describe the emergence of HAdV-7 in Malaysia. The outbreak occurred during March–April 2011 and involved new police recruits in the Kuala Lumpur Police Training Centre. Approximately 100 trainees were admitted to the Kuala Lumpur Hospital, and 4 more were treated in the intensive care unit. This outbreak affected 851 police trainees and claimed 3 lives.

## The Study

In April 2011, the virology unit at the Institute for Medical Research, Kuala Lumpur, received respiratory samples from police trainees admitted to Kuala Lumpur Hospital with ARD and tissue samples from 2 of the 3 patients with fatal cases. The postmortem specimens consisted of cerebrospinal, pericardial effusion, and pleural effusion fluids; lung, liver, spleen, and kidney tissues; skin; and bone marrow aspirate.

Viral nucleic acid was extracted from the clinical samples by using Roche High Pure Viral Nucleic Acid Kit (Roche Applied Science, Mannheim, Germany). Of the initial samples, 10 were screened for respiratory syncytial viruses, influenza viruses, parainfluenza viruses, human metapneumoviruses, coxsackieviruses, echoviruses, rhinoviruses, coronaviruses, adenoviruses, and bocavirus by multiplex PCR. All samples were then subjected to adenovirus nucleic acid detection by PCR.

Partial HAdV hexon gene was amplified by PCR (*8*). The SeqMan and Megalign software modules in the Lasergene suite of programs (DNASTAR, Madison, WI, USA) were used to format the nucleotide sequences. A phylogenetic tree was constructed by using the neighbor-joining method from the MEGA4 software (www.megasoftware.net).

A total of 33 clinical specimens, including respiratory and fecal samples as well as postmortem samples, from 21 trainees admitted to Kuala Lumpur Hospital were collected. Of these, only 31 samples from 19 trainees were sufficient for analysis. PCR or reverse transcription PCR was performed on 10 of the 31 samples by using primer sets specific for respiratory viruses with the ResPlex II Panel (QIAGEN, Valencia, CA, USA). The 10 samples contained HAdV-B species.

**Figure 1:**
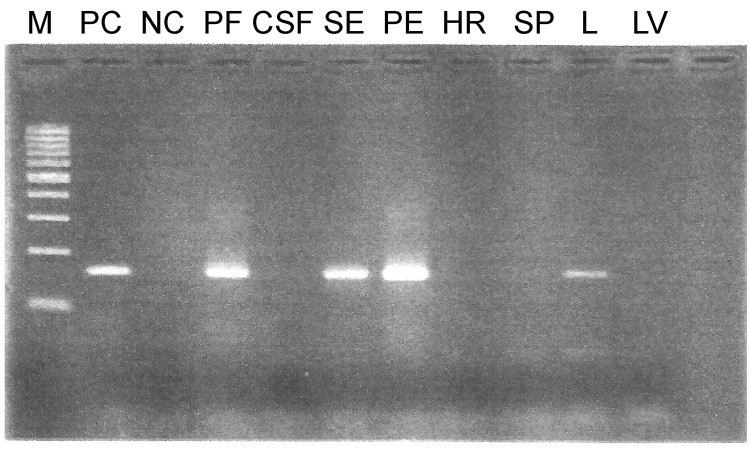
PCR product of postmortem samples RP381/11 on 3% agarose gel in study of human adenovirus type 7 outbreak in a police training center, Malaysia, 2011. M, 100-bp ladder; PC, positive control; NC, negative control; PF, pericardial fluid; CSF, cerebrospinal fluid; SR, serum; PE, pleural effusion; HR, heart; SP, spleen; L, lung tissue, LV, liver.

On the basis of this finding, partial hexon genes of adenovirus were amplified from all 31 samples by using PCR with HAdV-specific primers (*8*). The results (Table) indicated that 53% (10/19) of the samples tested were positive for adenovirus. An antemortem tracheal aspirate sample, received from the first case-patient who died, was positive for adenovirus. Subsequently, postmortem lung and spleen tissue samples tested by PCR were also positive for adenovirus. In the second fatal case, adenovirus was detected from postmortem samples consisting of pleural effusion fluid, pericardial effusion fluid, lung tissue, and serum, but other tissues, such as heart, spleen, and liver, and cerebrospinal fluid were negative (Figure 1).

**Table:** Patient information and adenovirus PCR results on samples from police trainees with acute respiratory disease from Kuala Lumpur, Malaysia, 2011*

Patient	Age, y†	Sample type	PCR results
RP301/11‡	22	T/A	Pos
RP302/11‡	25	T/A, antemortem	Pos
		Lung, spleen	Pos
		Kidney, liver, skin	Neg
		CSF, BMA	Neg
RP303/11	21	T/S	Pos
RP304/11	23	T/S	Pos
RP305/11	22	T/S	Pos
RP306/11	22	T/S	Neg
RP307/11	23	T/S	Neg
RP308/11	22	T/S	Neg
RP309/11	26	T/S	Neg
RP310/11	24	T/S	Pos
RP311/11	24	T/S	Pos
RP312/11	22	T/S	Pos
RP313/11	23	T/S	Neg
RP314/11	26	Feces	Neg
RP315/11	20	Feces	Neg
RP316/11	25	Feces	Neg
RP317/11	23	R/S	Neg
RP318/11	22	R/S	Pos
RP319/11	23	Feces	ND
RP320/11	20	Feces	ND
RP381/11‡	25	Lung, PF, PE, serum	Pos
		CSF, heart, spleen	Neg
		Liver	Neg

*T/A, tracheal aspirate; pos, positive; neg, negative; CSF, cerebrospinal fluid; BMA, bone marrow aspirate; T/S, throat swab; R/S, rectal swab; ND, test not done; PF, pericardial fluid; PE, pleural effusion. †All patients were male. ‡Fatal case.

**Figure 2:**
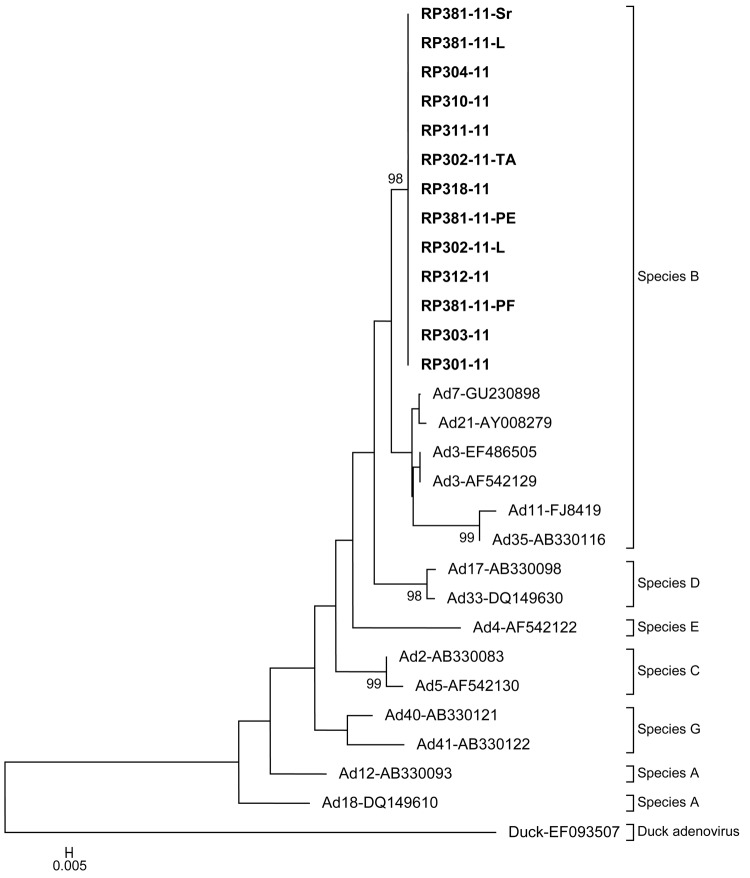
Phylogenetic tree of partial hexon gene sequences (160 bp) of human adenovirus inferred by using the neighbor-joining method from the MEGA4 software (www.megasoftware.net). Study was in a police training center, Kuala Lumpur, Malaysia, 2011. The evolutionary distances were computed by using the maximum composite likelihood method. Species A–F are indicated by square brackets with duck adenovirus A as an outgroup. Thirteen human adenovirus isolates from the police training center outbreak are indicated in **boldface**. Representative strains of each species obtained from GenBank are labeled by using the adenovirus species and accession number. Bootstrap values (>75%) for 1,000 pseudoreplicate datasets are indicated at branch nodes.

All positive samples were sequenced, and BLAST sequencing analysis (http://blast.ncbi.nlm.nih.gov/Blast.cgi) showed that their sequences were similar to human adenovirus type 7 strain 0901H2/Shix/CHN/2009 isolated in People’s Republic of China in 2009 (*9*). The phylogenetic tree, constructed on the basis of partial hexon gene (160) nucleotides (Figure 2), revealed that all positive samples by PCR belong to the species *Human adenovirus B* and are in the same cluster with adenovirus 7.

## Conclusions

The outbreak of ARD, caused by HAdV-7, in the Kuala Lumpur Police Training Centre started in early March 2011. The police trainees had signs and symptoms of ARD such as fever, cough, and loss of appetite. The disease rapidly spread among the trainees, and community-acquired pneumonia was the initial diagnosis early in the outbreak. Other etiologic agents that recently caused ARD, including seasonal influenza virus and pandemic (H1N1) 2009 virus, were excluded because the reverse transcription PCR for influenza viruses was negative.

The source of the infection is not known. There is a strong possibility that one of the trainees was infected with HAdV-7 in the community outside the training center and then spread the infection to others once training resumed. Transmission occurs through respiratory droplets and close contact, which leads to rapid and widespread dissemination. Similar events have been seen in military camps (*4*), high schools, and day care centers (*10*). All affected trainees were 20–26 years old. Risk factors such as overcrowding, increased physical activities, and psychological stress could possibly increase susceptibility to ARD. These risk factors associated with outbreaks of HAdV-caused ARD, which are prevalent in military and police training centers, have been reported in previous HAdV outbreaks in military recruits (*11*).

In Malaysia, 2 researchers (*12**,**13*) described the role of HAdV in causing ARD (*12*). They analyzed 27 HAdV isolates from patients with ARD who sought consultation and treatment at University of Malaysia Medical Center during 1999–2005. Among the 27 isolates, the following species were represented: 19 (70%) belonged to HAdV-C, 6 (22%) belonged to HAdV-B, and 2 (7%) belonged to HAdV-F. Among the HAdV-B species isolates, 5 had the HAdV-3 serotype and only 1 had the HAdV-7 serotype. An earlier analysis of HAdV isolates in Malaysia (*13*) revealed that HAdV-21 was associated with acute flaccid paralysis during the outbreak of hand, foot, and mouth disease in Sarawak, but none of the isolates were HAdV-7.

Pneumonia caused by HAdV-7, commonly associated with lower respiratory tract infection, can lead to severe disease and death in some infants and immunocompromised persons (*9*,*14*). Outbreaks of pneumonia caused by HAdV-7 have been reported among hospitalized children in South Korea and in the United States (*15*). This outbreak demonstrated the potential of ARD caused by HAdV-7 to produce illness and death in police and army recruit camps and in institutional settings. HAdV-7 infection also is fatal in children.
